# Supplementary material for: Near-infrared observations of active asteroid (3200) Phaethon reveal no evidence for hydration
Source: Nat Commun. 2020 Apr 28;11:2050. doi: 10.1038/s41467-020-15637-7 (PMC7188859; doi:10.1038/s41467-020-15637-7)
Supplement: Supplementary file 2 — Description of Additional Supplementary Files [file 41467_2020_15637_MOESM2_ESM.pdf]

Description of Additional Supplementary Files

File Name: Supplementary Data 1
Description: The processed (thermally, binned, and normalized) data of Phaethon that we used in the paper.

File Name: Supplementary Data 2

Description: The non-processed data of Phaethon that we used in the paper.
